# Supplementary material for: Trends of community-based systemic antibiotic consumption: Comparative analyses of data from Ethiopia and Norway calls for public health policy actions
Source: PLoS One. 2021 May 14;16(5):e0251400. doi: 10.1371/journal.pone.0251400 (PMC8121293; doi:10.1371/journal.pone.0251400)
Supplement: S1 Fig — (DOCX) [file pone.0251400.s001.docx]

●

●

●

●

●

*y*

=

0.2

+

0.033

*x*

*R*

2

=

0.89

0.20

0.25

0.30

0.35

2016

2017

2018

2019

2020

Years

Antibiotics consumption (log−transformed)

**S1 Fig. Log-linear regression model for systemic antibiotics consumption (DID) over a time period (2016-2020) in Ethiopia.**

Interpretation of the log-linear regression model:

1. The annual consumption of systemic antibiotics increased by 3.4% for each year between 2016 and 2020, on average. This is calculated from the log-linear regression fit as (exp(0.033)-1)•100, where 0.033 is the coefficient of the log-linear regression equation. Note also that the effect of year was significant as indicated by a p value = 0.016<0.05.
2. In the log-linear regression model, the correlation between the dependent variable (systemic antibiotics consumption, in DID) and the independent variable (time period, in years) is very strong (R^2^ = 0.89) — meaning, 89 % of variation in antibiotic consumption during the study period was explained by the differences in years.
