# Supplementary material for: Trends of community-based systemic antibiotic consumption: Comparative analyses of data from Ethiopia and Norway calls for public health policy actions
Source: PLoS One. 2021 May 14;16(5):e0251400. doi: 10.1371/journal.pone.0251400 (PMC8121293; doi:10.1371/journal.pone.0251400)
Supplement: Attachment — Submitted filename: Response to reviewer.docx [file pone.0251400.s002.docx]

**Response to the reviewer**

First of all, we would like to thank the reviewer for his/her constructive comments on the manuscript and also for the bold appreciation and encouragement extended to us on the time-consuming job of manually collecting the data from Ethiopia, doing the calculations and the analyses that we presented in our study.

Below, we give our responses/reflections on the reviewer’s comments:

**Reviewer’s comment**: “Pages 16-17: the font is bigger than in previous and later text.”

**Our response**: The reviewer is right. We have accordingly corrected this in the revised manuscript.

**Reviewer’s comment: “**Page 18 “[J01XA)” - wrong bracket”.

**Our response**: The reviewer is right. We have accordingly corrected this in the revised manuscript.

**Reviewer’s comment**: “From more important things...The introduction require data from Norway and Ethiopia about use of antibiotics (one paragraph). I notice that you mention that “Data on annual antibiotic consumptions in public health sectors are not publicly available for Ethiopia” but maybe there are some information about use of antibiotics in agriculture and in medicine, types of antibiotics or at least situation in Africa.”

**Our responses/reflections**: The reviewer is correct in recommending to include data about antibiotic use in Ethiopia and Norway in the introductory section of our manuscript – we noted that with compliments. We also noted reviewer’s acknowledgement about lack of studies on Ethiopia’s country-level consumption of antibiotics with compliments. There are however a few health facility-based studies published on antibiotic consumption and resistance, including one co-authored by one of the co-authors in this manuscript. We therefore have added one paragraph to the introductory part, as per the recommendation, citating those studies from Ethiopia and also the required country-level consumption data from Norway using references from 16 to 21. We therefore have corrected this, too, in the revised manuscript, accordingly.

**Reviewer’s comment:** “Results and Discussion should be written separately - this is my major concern. Discussion should be a separate paragraph and must be enhance in literature.”

**Our response:** If taken with respect to PLOS ONE’s manuscript submission guidelines on “[Manuscript Organization … Results, Discussion](https://journals.plos.org/plosone/s/submission-guidelines#loc-results-discussion-conclusions)...”, which we did consult before submitting our article, the reviewer might not be correct because the submission guidelines offer an option to either put “Results and Discussion” together or present them separately. However, in line with the reviewer's recommendation, we have accordingly re-written the results and discussion separately in the revised version of our manuscript.

**Sincerely**

Authors
